# Illumina short-read sequencing data, *de novo* assembly and annotations of the *Drosophila nasuta nasuta* genome

**DOI:** 10.1016/j.dib.2020.106674

**Published:** 2020-12-19

**Authors:** Stafny DSouza, Koushik Ponnanna, Amruthavalli Chokkanna, Nallur Ramachandra

**Affiliations:** Department of Studies in Genetics and Genomics, University of Mysore, Mysuru, India

**Keywords:** *Nasuta* subgroup, Genome assembly, Illumina sequencing, Annotation, Genome evolution

## Abstract

The *Drosophila nasuta nasuta* (*D. n. nasuta*) is a member of *nasuta* subgroup of *immigrans* species group of *Drosophila* widely distributed across South-East Asia and central to Southern Africa. It displays morphological similarities with other members of the *nasuta* subgroup with which it has a recent divergence history. The genomic DNA of *D. n. nasuta* Coorg strain was paired-end sequenced using Illumina HiSeq 2500 technology to obtain a draft genome assembly of 145.64 Mb. The generated assembly retrieved 93.6% of the conserved dipteran BUSCO orthologs. Approximately 85% of the *ab initio* predicted proteins exhibit sequence similarity to the proteins of *D. albomicans* which is the closest annotated species. This draft genome sequence is a valuable resource to *Drosophila* geneticists and evolutionary biologists to understand molecular organisation of the genome and its evolution during early stages of speciation.

## Specifications Table

**Table:** 

Subject	Insect science
Specific subject area	Bioinformatics (Genomics)
Type of data	Tables Chart Graphs
How data were acquired	The data was acquired by Next-Generation Sequencing technology using lllumina HiSeq 2500 and draft assembly was generated by Platanus v1.2.4, SSPACE-Standard v3.0 and GapCloser v1.0.
Data format	Raw analysed
Parameters for data collection	Genomic DNA was extracted from 40 whole males using MP Biomedicals FastDNA™ SPIN Kit. Raw high-quality reads were generated through Illumina paired-end sequencing and assembled through *de novo* method.
Description of data collection	*D. n. nasuta* flies were maintained at Department of Studies in Genetics and Genomics fly facility, University of Mysore, India. Male flies were isolated and aged for 5 days. Genomic DNA extracted from whole male flies, following sequencing, draft genome assembly and annotation
Data source location	Institution: University of Mysore City/Town/Region: Mysuru, Karnataka Country: India Latitude and longitude (and GPS coordinates, if possible) for collected samples/data: 12°18′59.9″N 76°37′14.5″E
Data accessibility	Raw Illumina sequence data is deposited in NCBI-SRA (Short Read Archive) repository with accession number SRX8655761 and can be accessed using the URL https://trace.ncbi.nlm.nih.gov/Traces/sra/?run=SRR12134549 This Whole Genome Shotgun project has been deposited at DDBJ/ENA/GenBank under the accession JADFWM000000000. The version described in this paper is version JADFWM 010,000,000. All additional data analysis files can be accessed from Mendley Data using the link http://dx.doi.org/10.17632/3s7tft6vgb.3

## Value of the Data

• This data is a valuable resource to *Drosophila* geneticists and evolutionary biologists studying genetics and species divergence.
• Draft genome of *D. n. nasuta* will provide useful insights into understanding the mechanisms underlying genome evolution contributing to speciation.
• The *D. n. nasuta* genome sequences when compared to its recently-diverged sibling species, can aid in understanding the mechanisms associated with whole chromosome fusions and their maintenance.
• This genome will form a tractable genomic system for large-scale evolutionary experimentation on raciation and speciation as it constitutes an artificial hybrid zone in the environs of the laboratory with its sibling species *D. n. albomicans* and their fertile laboratory hybrid lineage called Cytoraces.

## 1 Data Description

Initial steps towards understanding the genetics of speciation includes the genomic characterization of the study target. To this end, we sequenced the genome of *D. n. nasuta* by Illumina paired-end sequencing technology. *D. n. nasuta* is a member of *nasuta* subgroup of *immigrans* species group belonging to genus *Drosophila*. The *nasuta* subgroup, which harbors several closely related species/ sub-species pairs exhibiting striking morphological similarities with varying degrees of reproductive isolation [1]. *D. n. nasuta* has a recent divergence history (0.3- 0.6 million years) with its sibling species *D. n. albomicans*, with which it can produce fertile hybrids under laboratory conditions [2]. The major difference between the two species is marked by fusion of the 3rd autosomes and sex chomosomes in *D. n. albomicans* [1,3]. Hence, this draft genome can help in understanding mechanisms of whole chromosome fusion and their maintenance during the early stages of divergence. Here, we present a high quality Illumina draft genome assembly for *D. n. nasuta* (Coorg strain, Mysore) with better N50 values and contiguity than the existing asembly [4] of samples collected from different geographical location.

**Fig. 1:**
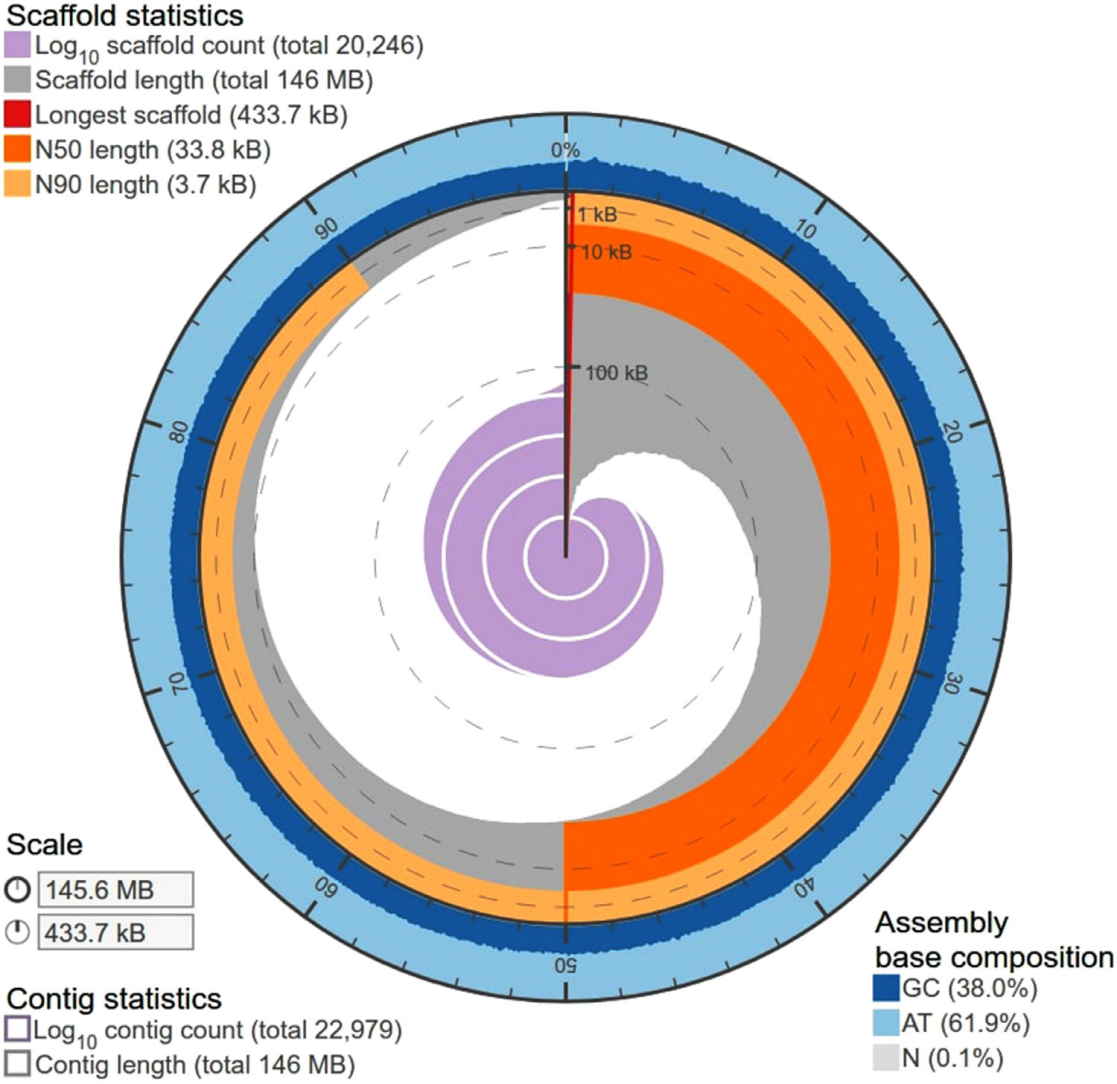
Circular plot visualizing the assembly metrices of the final *D.n. nasuta* draft genome assembly.

**Fig. 2:**
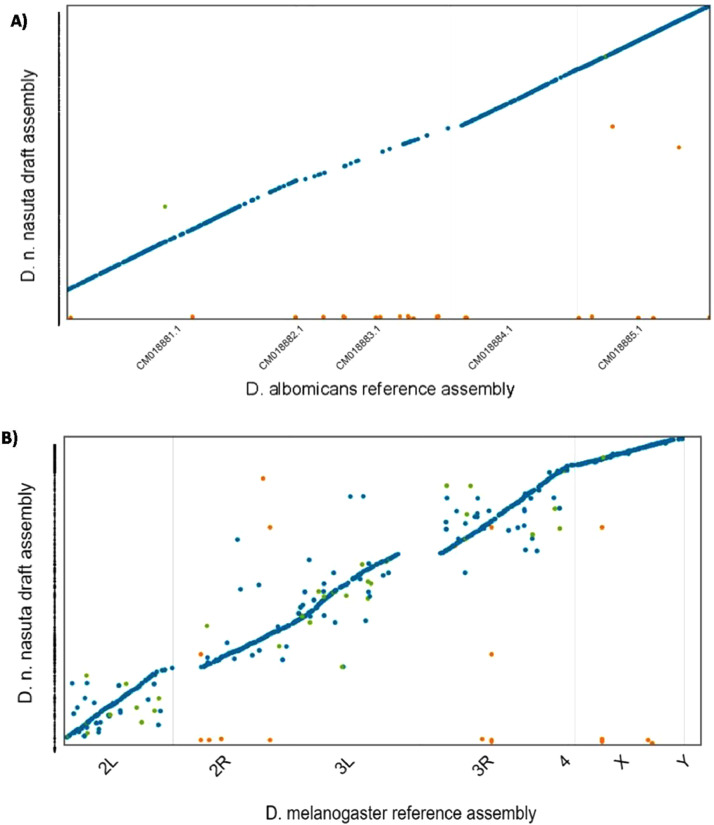
Dotplot showing alignment of the assembled *D. n. nasuta* draft assembly scaffolds (along Y- axis) to the chromosomes in (A) *D. albomicans* reference assembly (B) *D. melanogaster* reference assembly. The coloured dots represent unique forward alignments (blue), unique reverse alignments (green) and repetitive alignments (orange). (For interpretation of the references to color in this figure legend, the reader is referred to the web version of this article.)

**Fig. 3:**
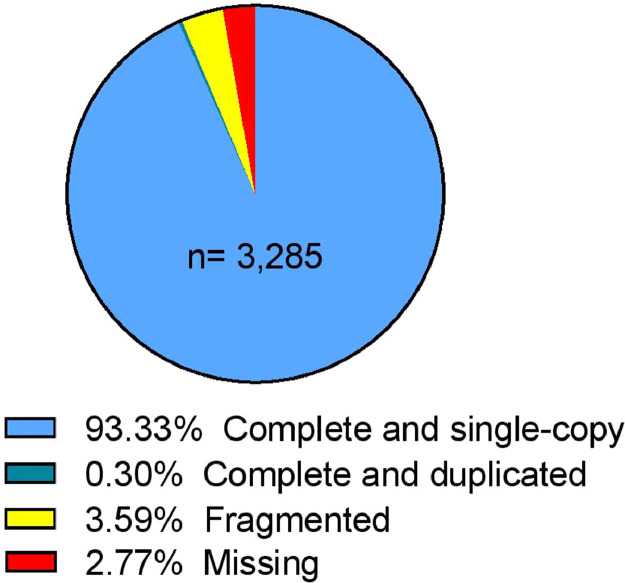
Schematic representation of results from Benchmarking Universal Single-Copy Ortholgs (BUSCO) analysis for genome assembly completeness. Colours indicate degrees of completeness of the predicted genes in the assembly. ‘n’ indicates the total number of genes in *diptera_odb10* dataset.

Paired-end Illumina sequencing of *D. n. nasuta* gDNA generated 7.5 Gb of sequence data. Filtering of Illumina adapters and low-quality sequences removed 0.037% of the 137.4 million paired-end raw reads. Read statistics are provided in Table 1. A total of 137.3 million high quality filtered 2 × 101 bp reads were initially assembled into 73,720 ungapped contigs. Scaffolding, gapfilling and NCBI check of the contig assembly produced a final draft asembly of 145.64 Mbp consisting of 20,246 scaffolds. Assembly statistics are presented in Fig. 1 and Table 2. About 79.15% of the high quality filtered reads mapped back to the assembled draft genome. Collinearity between the draft assembly of *D. n. nasuta* and closely related *D. albomicans* genome and distantly related *D. melanogaster* genome is illustrated in Fig. 2. For further analysis, only 11,950 scaffolds with minimum length of 1000 bp were considered. The Benchmarking Universal Single-Copy Ortholgs (BUSCO) analysis on these scaffolds predicted the presence of 3066 complete single copy conserved eukaryotic genes out of the 3285 known genes in *diptera_odb10* dataset. Overall, 93.6% of the predicted genes were complete (Fig. 3).

**Table 1:** Read statistics of *D. n. nasuta* Illumina sequencing.

		Raw reads	Filtered reads
Read statistics	Read count	137,416,614	137,364,956
	No. of bases (bp)	13,879,078,014	13,833,421,519
Nucleotide percentages (%)	A	35.055	35.065
	T	34.915	34.925
	G	15.02	15
	C	15.01	15
	N	0.0045	0.0025
Dinucleotide percentages (%)	AT	69.97	69.995
	GC	30.03	30.005

**Table 2:** Statistics for various stages of *de novo* assembly construction of *D. n. nasuta*.

	Initial contig assembly	After contigs reduction	After scaffolding	Final Draft assembly
Assembled bases (bp)	148,583,738	144,437,007	145,192,980	145,640,604
Number of contigs/ scaffolds	73,720	59,504	20,275	20,246
N50 (bp)	10,508	11,586	33,806	33,794
GC Content (%)	37.01	37.96	37.94	38.0
Largest contig/scaffold size (bp)	248,159	248,159	433,436	433,675
Total number of N's	0	0	837,739	111,412

About 18,881,583 bp (13.27%) of the assembled sequences were softmasked as repetitive sequences by RepeatMasker. A total of 15,283 gene models were predicted by AUGUSTUS of which, 4483 had >90% exon evidence. The genes predicted by other *ab initio* gene predictors are summarised in Table 3. An input of all 83,966 gene models to EvidenceModeler (EVM) resulted in retrieval of 15,432 total gene models. After filtering of bad gene models containing gaps, transposable elements and shorter protein length (<100 amino acids), 13,766 protein coding genes were finally retained. The tRNAScan-SE tool identified 222 tRNA coding genes in the assembly.

**Table 3:** Summary of gene prediction for *D. n. nasuta* draft genome assembly.

Tool	Prediction type	Total genes
Trinity- PASA	Predicted genes	9260
AUGUSTUS	Predicted genes	10,800
AUGUSTUS	HiQ Predicted genes	4483
GeneMark-EP+	Predicted genes	15,421
GlimmerHMM	Predicted genes	18,458
SNAP	Predicted genes	25,544
EvidenceModeler	Predicted genes	13,768
tRNAScan-SE	Predicted tRNA genes	222
	Final protein coding genes	13,766

HiQ- High quality ( type="Dataset">90% exon evidence).

**Fig. 4:**
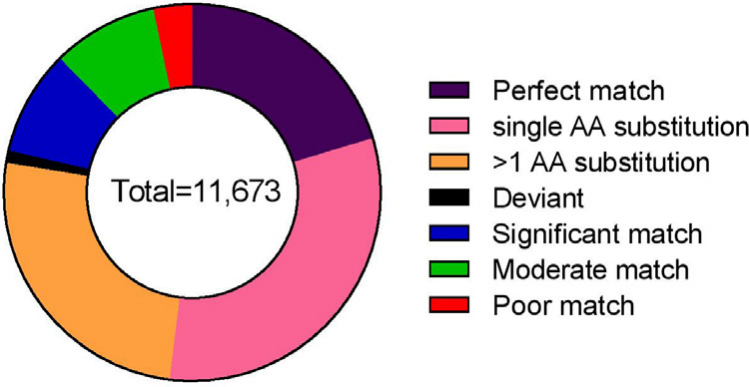
Annotation integrity check of the *D. n. nasuta* proteins from BLASTp against the *D. albomicans* proteins. The annotation integrity was classified into different categories considering the query coverage (Qcov) and protein similarity (Sim) as: Perfect match (Qcov= 100%; Sim= 100%), single amino acid (AA) substitution (Qcov= 100%; Sim >99%), >1 AA substitution (Qcov= 100%; Sim >90%), Deviant (Qcov <100%; Sim <80%), Significant match (Qcov <100%; Sim >97%), moderate match (Qcov <100%; Sim 80–97%), poor match (Qcov <100%; Sim <80%).

**Fig. 5:**
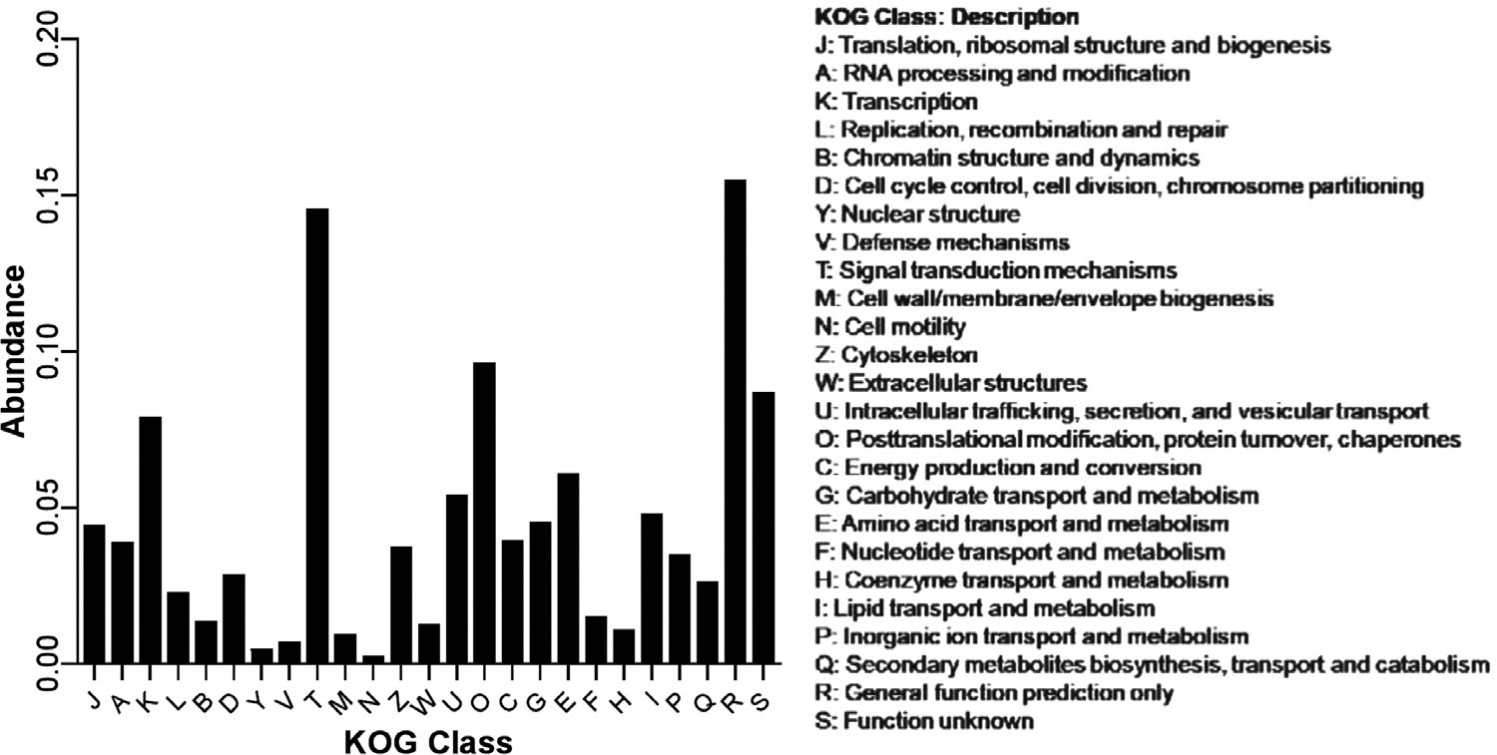
An overview of Eukaryotic Orthologous Group (KOG) classification of *D. n. nasuta* proteins.

**Fig. 6:**
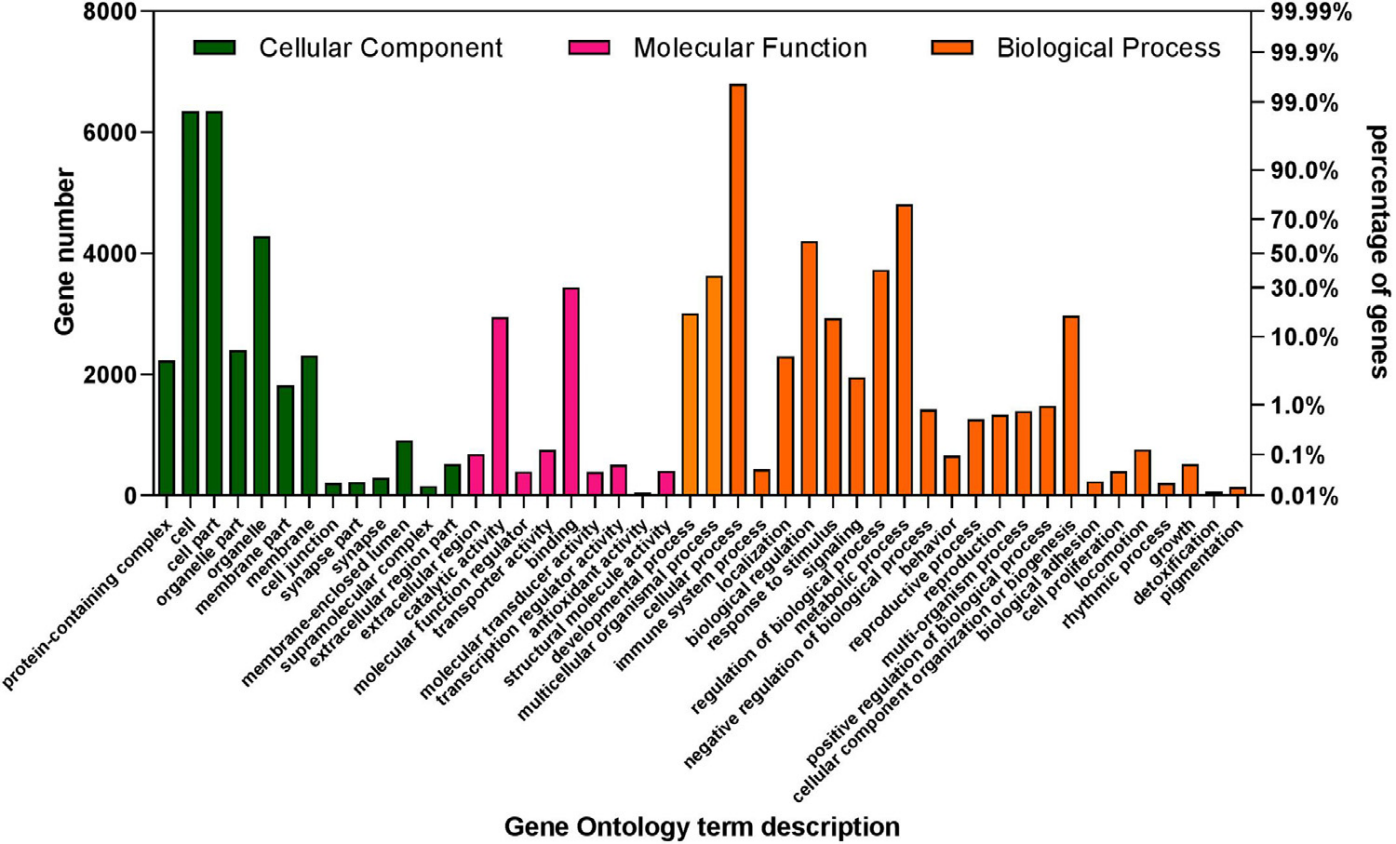
The Gene Ontology (GO) annotation of *D. n. nasuta* proteins classified as Cellular component (green), Molecular functions (pink) and Biological processes (orange). (For interpretation of the references to color in this figure legend, the reader is referred to the web version of this article.)

11,673 out of the 13,766 predicted protein sequences annotated against *D. albomicans* proteins*.* 9353 (80%) of the annotated proteins were represented by nearly full-length transcripts having a protein alignment coverage of >80%. The distribution of sequence similarity of the annotated protein at different query coverage (percentage of the annotated protein length included in the BLASTp alignment) intervals is shown in Fig. 4. The KOG class distribution is shown in Fig. 5. The data illustrated in Fig. 6 shows the Gene Ontology (GO) distribution of the protein coding genes.

## 2 Experimental Design, Materials and Methods

### 2.1 Fly stock and DNA extraction

The *Drosophila nasuta nasuta* (Coorg strain, India; Stock number: 201.001, Drosophila Stock Centre, University of Mysore) was maintained on wheat cream agar media at 22±1°C temperature, 60% humidity and 12hr light/dark cycle at the Department of Studies in Genetics and Genomics fly facility. Male flies were isolated on eclosion and aged for 5 days. Genomic DNA (gDNA) was extracted from 40 whole males using MP Biomedicals FastDNA™ SPIN Kit following the manufacturer protocol. Thermo Scientific™ NanoDrop 2000 spectrophotometer and Qubit™ Flex Fluorometer (dsDNA BR Assay) checked the quality and quantity of gDNA.

### 2.2 Genome sequencing, quality assessment and de novo assembly

Paired-end (PE) libraries were generated from the quality checked (QC) gDNA using Illumina True-Seq Nano DNA library preparation kit as per manufacturer's specifications and validated using Agilent DNA HS and Qubit™ DNA BR. The QC passed libraries were sequenced on Illumina HiSeq® 2500 platform (Illumina, Inc., USA) for 2 × 101 bp read length at InterpretOmics India Private Ltd., Bengaluru, India. Raw reads were processed to trim adaptor sequences and low quality regions using *Platanus_trim* and *de novo* assembled with Platanus (v1.2.4) [5]. Redundans (v0.11-beta) [6] removed the redundant contigs from this initial assembly. The non-redundant contigs were then scaffolded by SSPACE-Standard (v3.0) using the high-quality paired-end data [7] and gap-filled by GapFiller (v1.1.0) [8] to build a draft assembly. The final draft assembly is accessible from Genbank (accession: JADFWM000000000). Assembly statistics were generated by assembly-stats tool [9]. The quality of the draft assembly was assessed by mapping the filtered reads to the draft genome using minimap2 [10] and BUSCO (v4.0.5) analysis using the *diptera_odb10* lineage dataset [11]. The *D. n. nasuta* draft genome assembly was also aligned to its closely related *D. albomicans* reference assembly (accession: GCF_009650485.1) and distantly related *D. melanogaster* reference assembly (accession: GCF_000001215.4) using MUMmer3’s nucmer[12]. Alignment plots were generated using Dot tool [13].

### 2.3 Structural and functional annotations

To maximize gene predictions, the repeat elements in the assembly were masked by RepeatMasker (v4.1.0; http://www.repeatmasker.org) using a custom repeat library constructed using RepeatModeler (v2.0.1) [14]. For structural genome annotations, a training dataset was generated by *funannotate train* function in Funannotate (v1.8.1) [15] using the RNA-seq reads from gonadal tissues of *D. n. nasuta* Coorg strain (SRA accession numbers: SRR10875323 and SRR8398946). Briefly, RNA-seq reads were assembled by genome-guided module in Trinity. The predicted transcripts were then aligned to the softmasked genome to construct PASA gene models. Gene models were then generated by *funannotate predict* function in Funannotate. Briefly, using PASA gene models as training dataset, gene models were constructed by *ab initio* gene predictors like AUGUSTUS, GlimmerHMM and SNAP integrated in Funannotate. Additionally, gene models were also predicted by Genemark-EP+ [16]. EvidenceModeler then combined all *ab initio* gene model predictions along with protein evidence from UniprotKB/Swissprot database and *D. albomicans* proteins to generate a final set of protein coding genes. Finally, tRNAscan-SE [17] validated the tRNA coding genes.

The predicted protein coding sequences were annotated against the known *D. albomicans* proteins using the BLASTp program [18] with an e-value of 0.00001. The annotation integrity was categorised at three query coverage (Qcov) intervals with protein similarities (Sim) as: Perfect match (Qcov= 100%; Sim= 100%), single amino acid (AA) substitution (Qcov= 100%; Sim >99%), >1 AA substitution (Qcov= 100%; Sim >90%), Deviant (Qcov= 100%; Sim <80%), Significant match (Qcov <100%; Sim >97%), moderate match (Qcov <100%; Sim 80–97%), poor match (Qcov <100%; Sim <80%). The Gene Ontology (GO) terms for the predicted proteins were extracted based on protein similarity to UniprotKB/swissprot database (Release 2019_11). KOG annotations were performed on webMGA server [19] with e-value of 0.00001. The parameters and data files of all analyses performed can be accessed from Mendley Data [20]

## Declaration of Competing Interest

The authors declare that they have no known competing financial interests or personal relationships which have, or could be perceived to have, influenced the work reported in this article.
